# Building a biomedical tokenizer using the token lattice design pattern and the adapted Viterbi algorithm

**DOI:** 10.1186/1471-2105-12-S3-S1

**Published:** 2011-06-09

**Authors:** Neil Barrett, Jens Weber-Jahnke

**Affiliations:** 1 Department of Computer Science, University of Victoria, Victoria, Canada

## Abstract

**Background:**

Tokenization is an important component of language processing yet there is no widely accepted tokenization method for English texts, including biomedical texts. Other than rule based techniques, tokenization in the biomedical domain has been regarded as a classification task. Biomedical classifier-based tokenizers either split or join textual objects through classification to form tokens. The idiosyncratic nature of each biomedical tokenizer’s output complicates adoption and reuse. Furthermore, biomedical tokenizers generally lack guidance on how to apply an existing tokenizer to a new domain (subdomain). We identify and complete a novel tokenizer design pattern and suggest a systematic approach to tokenizer creation. We implement a tokenizer based on our design pattern that combines regular expressions and machine learning. Our machine learning approach differs from the previous split-join classification approaches. We evaluate our approach against three other tokenizers on the task of tokenizing biomedical text.

**Results:**

Medpost and our adapted Viterbi tokenizer performed best with a 92.9% and 92.4% accuracy respectively.

**Conclusions:**

Our evaluation of our design pattern and guidelines supports our claim that the design pattern and guidelines are a viable approach to tokenizer construction (producing tokenizers matching leading custom-built tokenizers in a particular domain). Our evaluation also demonstrates that ambiguous tokenizations can be disambiguated through POS tagging. In doing so, POS tag sequences and training data have a significant impact on proper text tokenization.

## Background

Natural language processing (NLP) is the computer processing of human language [1]. It is a bidirectional chain of transformation from speech to language understanding - from sounds to semantics. Segments of this processing chain are designed to address different NLP problems, including audio to text transformation, text processing and semantic recognition. This paper focuses on text processing.

Tokenization typically plays a role in processing text. Tokenization is broadly defined as the segmentation of text for subsequent processing. The definition’s breadth reflects the ambiguity and differences of tokenization strategies. Tokenization strategies can vary depending on language [2,3], software goals [4] and other criteria. There is no widely accepted tokenization method for English texts, including biomedical texts [2,4-7].

In contrast, there are widely accepted solutions to other NLP tasks. The Viterbi algorithm is a widely accepted solution for part-of-speech (POS) tagging [1]. POS tagging assigns tags to tokens, such as assigning the tag *Noun* to the token *paper*. Similarly, the CKY algorithm is a widely accepted solution for syntactic parsing [1]. Syntactic parsing constructs a syntactic structure such as a parse tree from a sequence (e.g. sentence) of tagged tokens.

Although there is no widely accepted tokenization method, tokenization is an important component of language processing [2,8-10]. As Webster and Kit [2] argue, tokenization identifies basic units on which further processing depends. For example, tokenization segments a sentence’s terminating symbol from its last word allowing subsequent processing to identify a text’s sentences (e.g. “He wrote a paper.” becomes “He wrote a paper .”, tokenization of “paper.” to “paper .”).

Hassler and Fliedl [11] suggest that tokenization is often perceived as a solved problem. For Tomanek, Wermter and Hahn [5], tokenization can be perceived as “unsophisticated clerical work”. On the other hand, there is evidence to support that tokenization is not trivial. A single Arabic word can be composed of four independent tokens [3]. Chinese words do not have obvious boundary markers [2]. Spanish and English can be considered to flow across whitespace boundaries (e.g. sin embargo [12] and New York). Biomedical names pose tokenization difficulties because they often contain special characters such as slashes or brackets [4]. Proper tokenization in these contexts is a non-trivial problem [2,4-6,9,13].

Within the domain of biomedical tokenization, He and Kayaalp [7] applied 13 tokenizers to 78 MEDLINE abstracts. Only 3 of the 13 tokenizers produced identical results and the differing results varied widely. Given the latter, He and Kayaalp advocate awareness of a tokenizer’s details without clearly defining or specifying which tokenizer details are important. Tokenizer details are expected to influence whether a tokenizer is well suited or adaptable to a particular language processing task. A poor choice of tokenizer is expected to cause (unintentional) information loss [7].

Several tokenizers examined by He and Kayaalp [7] used simple rule based tokenization methods (e.g. regular expressions). Jiang and Zhai’s [4] empirical study of rule based tokenization supports the use of rule based tokenizers on specific texts. Rule based tokenization methods may perform well for specific texts but these methods appear to generalize poorly [4,7].

Other than rule based techniques, tokenization in the biomedical domain has been regarded as a classification task [5,6,13,14]. Classification assigns a label to objects. For example, a classifier could assign a token-separator label to the space character. Classification tokenizers differ in their choice of object and their method for learning and applying tags.

Biomedical classification-based tokenization can be divided into two approaches: classifiers that classify textual objects as a token boundaries (or not) and classifiers that reassemble primitive tokens. In other words, classifier-based tokenizers either split or join textual objects through classification. Split-join based tokenization approaches have applied a variety of machine learning methods with success as exemplified below.

A classifier was used to label selected symbols such as a space or a period as within a token or as a token separator [6]. This *split* approach performed well on named entity only data (e.g. person, organization) and poorly on named entities in MEDLINE abstracts. This approach neglects un-delimited tokens such as “2.5cm”.

McDonald, Crammer and Pereira [14] applied multi-label classification techniques to tokenization. Their classifier assigned beginning (B), inside (I) and outside (O) labels to primitive token sequences. The segments labeled with a B followed by consecutive I labels represented a single large token. This *join* approach might also be considered as *over-segment and repair* because their classifier reassembled incorrectly segmented tokens.

Tomanek, Wermter and Hahn [5] trained two (*split* approach) classifiers to identify sentence and token boundaries using a corpus derived from the PennBioIE and GENIA corpora. Input text was split into sentences and sentences were split into tokens. The token-splitting classifier used preset token boundary symbols and corpus-based training to identify token boundaries.

Wrenn, Stetson and Johnson [13] used transitional entropy and conditional probability to detect token boundaries (*split* approach). They compared their tokenization method to human specified sentence boundaries and a rule based tokenizer that segmented sentences by whitespace. The authors acknowledge that the lack of a gold standard is the most important limitation of their work. An example of this limitation is that their method is not evaluated on whether punctuation such as a comma is indicative of a token boundary.

### Motivation

We attempted to select an existing biomedical tokenizer for a biomedical text processing task. The idiosyncratic nature of each biomedical tokenizer’s output, or documented output, complicated our selection. He and Kayaalp [7] similarly found that output varied between tokenizers (recall that only 3 of the 13 tokenizers He and Kayaalp tested produced identical results). Furthermore, we found that existing biomedical tokenizers generally lacked guidance on how to apply the tokenizer to new text. As an example of the guidance we sought, consider the question of how improper tokenization of tokens, existing only in the new text, should be resolved.

To address the above difficulties, we identify and complete a novel tokenizer design pattern and suggest a systematic approach to tokenizer creation. In so doing, we provide a definition of tokenization and describe software components to accompany the proposed definition. We implement a tokenizer based on our design pattern that combines regular expressions and machine learning. Our machine learning approach differs from the previous split-join classification approaches. We evaluate our approach against three other tokenizers on the task of tokenizing biomedical text.

## Results

### Algorithm and Implementation

In this section, we present a novel tokenizer design pattern for biomedical tokenizers. According to Buschmann, Henney and Schmidt [15], “a design pattern provides a scheme for refining elements of a software system or the relationships between them. It describes a commonly-recurring structure of interacting roles that solves a general design problem within a particular context.”. We present our tokenizer design pattern by defining a tokenizer’s input and output, by defining a tokenizer’s software components and by presenting related pseudocode. Our tokenizer design pattern is named the *token lattice design pattern*.

#### Input and output

Current tokenizers generally compute on raw text (e.g. [13]) or sentences (e.g. [14]). We restrict a tokenizer’s input to raw text. If the text contains well formed sentences then it may be possible to use existing software that segments text into sentences with few errors (e.g. Punkt [16]).

A tokenizer’s output definition should communicate a tokenizer’s behaviour and foster tokenizer reuse. He and Kayaalp [7] discuss the variability in tokenizer output. Underlying this difference in output is a lack of agreement on what constitutes a token. Furthermore, tokenizers produce tokens based on an intrinsic token definition. Tokenizer output is generally idiosyncratic (e.g. format, token choices).

We restrict a tokenizer’s output to the most likely POS-tagged sequence of tokens, given some language model. This implies that a tokenizer outputs tokens taggable with tags such as noun or adjective. It also implies that a tokenizer must implement predefined POS tags such as the Penn Treebank’s [17]. Lastly, it implies that a tokenizer should produce a likely sequence of POS-tagged tokens. For example, a tokenizer should not segment a chemical substance such as “3,4-epoxy-3-methyl-1-butyl-diphosphate” into (space delimited) “3 , 4 epoxy 3 methyl 1 butyl diphosphate”. We define the concept of *POS-tokens* as tokens that adhere to our stated output restrictions. These restrictions blur the conventional boundary between tokenizers and POS-taggers (the tokenizer could easily tag tokens during tokenization). We argue below that POS-tokens are expected to increase tokenization accuracy and tokenizer reuse.

Chaining arbitrary tokens together is unlikely to form a valid (English) sentence. Accordingly, knowing a token’s POS tag indicates which POS tags and tokens are likely to occur in the token’s vicinity [1]. For example, it is likely that a noun follows after the word *the* (e.g. the hands), whereas it is less likely that a verb follows *the* (e.g. the wrote). POS-tokens inherit language characteristics that are likely to increase tokenization accuracy given that these characteristics have been successfully exploited in the past (e.g. Viterbi algorithm).

Inter-annotator agreement can be measured for POS tagging. This is a measure of agreement between people performing manual POS tagging of text. For example, the Penn Treebank’s inter-annotator agreement for POS tagging is above 90% [17]. Since algorithms can mimic human behaviour when assiging POS tags to tokens (e.g. [18]), tokenizers that output POS-tokens are expected to produce valid POS-token sequences and consequently mimic human performance. For example, two tokenizers adhering to Penn Treebank POS tags should segment sentences with over 90% agreement given individually successful implementations. POS-tokens should foster consistent human-like tokenization behaviour. Such behavior is expected to increase tokenizer reuse.

A tokenizer is a function that given some text and context segments the text into tokens. In our approach, the segmentation adheres to a language model and each token maps to a POS tag.

The notion of a tokenizer can be formalized as *T* := (Σ, *L_m_*, Γ)

• Σ is a finite set of symbols called the alphabet.

• *S* is the set of all finite strings over Σ and *S*′ := *S* + {*ε*}, includes the empty string.

• *L_m_* is a language model (e.g. a probabilistic model for parsing sentences) that includes a finite set of POS tags and a finite set of tokenization contexts.

• *E*(*L_m_*) := *E* is a finite set of POS tags.

• *C*(*L_m_*) := *C* is a finite set of contexts where a context is a tuple of information specific to a tokenizer instance. For example, a context could contain the previous sentence’s parse or simply the previous token.

• *T_t_* is the set of all tuples over *S* × *E.* These tuples represent sequences of tagged tokens, excluding empty tokens.

• Γ : *C* × *S*′ → *T_t_*

A good tokenizer is a tokenizer that chooses the most likely sequence of tagged tokens for a given context, input and language model. Thus, a good tokenizer satisfies:

• ∀*c ε C*, *s ε S*′ Γ(*c*, *s*) = *argmax t_t_εT_t_ P*(*t_t_*|*c*, *s*, *L_m_*).

• where argmax is (customarily) defined as a function that, given an expression resulting in a real-value and a set of elements, returns the subset of elements that maximize the expression’s value.

Our design pattern and guidelines are expected to create good tokenizers.

#### Components

**Figure 1:**
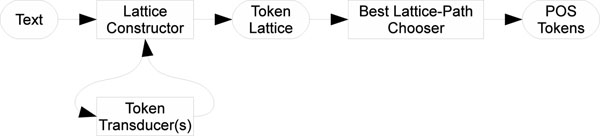
**Tokenizer components and information flow** A diagram illustrating the tokenizer’s components and information flow through these components.

Having defined a tokenizer’s input and output, we further define a tokenizer by defining its internal structure; its software components. We separate a tokenizer into three components: a token lattice and lattice constructor, a best lattice-path chooser and token transducers. Token transducers create candidate tokens from text. These candidate tokens are assembled into a token lattice by the lattice constructor. The best path (tokenization) is selected from the token lattice, tokenizing the text. These components are illustrated in Figure 1. The components are further explained below.

Text may have multiple segmentations caused by ambiguous token boundaries. For example, the sentence “The patient’s 10mg tablet.” segments into eight token sequences given that “patient’s”, “10mg” and “tablet.” could also be interpreted as (space delimited) “patient ’s”, “10 mg” and “tablet .”. The symbols ’ *m* and . ambiguously act as token boundaries in English (e.g. “tablet.” versus “2.3”).

**Figure 2:**
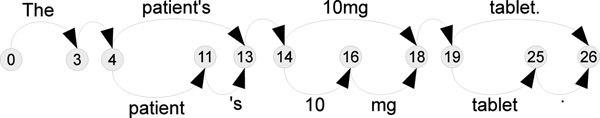
**A bounded lattice representing a sentence’s segmentations** An example of a bounded lattice representing a sentence’s segmentations.

A bounded lattice [19] can represent a text’s segmentations. In this context, a bounded lattice is a partially ordered set of segmentations with a least and greatest element (e.g. Figure 2). Such a lattice is referred to as a token lattice. Conceptualizing a sentence’s segmentations as a bounded lattice has been suggested previously [8,20,21], but has not been applied to biomedical tokenizers or biomedical text. It is unknown whether or not a token lattice is appropriate for biomedical tokenization. We formalize and complete the token lattice design pattern for the biomedical domain.

When converting text to a token lattice, it may be necessary to transform a text’s raw candidate tokens into candidate tokens that increase the text’s POS-tag (sequence) likelihood. For example, it may be necessary to transform the token “mg” into “milligrams” to increase the POS-tag likelihood of the sentence “The patient’s 10mg tablet.”. Increasing POS-tag likelihood is meant to satisfy our tokenizer definition, that of likely POS tag sequences.

Token transducers identify and transform a text into candidate token sequences for the token lattice. The candidate token sequences are inserted into the token lattice by the lattice constructor.

A token transducer is formally defined as follows:

*T_transducer_ :*= (Σ, *L_m_*, *τ*)

• Σ is a finite set of symbols called the alphabet.

• *S* is the set of all finite strings over Σ and *S*′ := *S* + {*ε*}, includes the empty string.

• *L_m_* is a language model (e.g. a probabilistic model for parsing sentences) that includes a finite set of tokenization contexts.

• *C*(*L_m_*) := *C* is a finite set of contexts where a context is a tuple of information specific to a tokenizer instance.

• *T_s_* is the set of all tuples over *S*. These tuples represent token sequences.

• *τ* : *C* × *S*′ → ℕ_0_ × *T_s_.* The transduce function returns the length of text used and a corresponding sequence of tokens.

Applying an implementation of the transduce function to the example string “10mg of” might result in: *τ_impl_*( null, “10mg of” ) = (4, (“10”, “milligrams”)). The transduce function’s output is restricted such that the quantity of text used by the transducer is bounded by the length of the input, *l ε* [0, *length*(*s*)], given (*l*, *t_s_*) *ε* ℕ_0_ × *T_s_* and some *s ε S*′. A value of (0, ∅) indicates that the transducer could not be applied.

The token transducer formalization assumes that the token transducer operates from the input string’s beginning. An alternate formalization includes an index into the input string specifying the location on which to apply the transducer.

To complete the tokenizer’s components, an algorithm is required that chooses the best path (tokenization) from the token lattice and one that constructs the token lattice from token transducer output. The token lattice’s best path is the most likely path through the token lattice given some language model. An algorithm exists for best path selection (e.g. adapted Viterbi [12]).

To construct a token lattice, a lattice constructor applies every transducer to each character position in the input text. The result of applying a lattice constructor on “The patient’s 10mg tablet.” is seen in Figure 2.

Given:

• Σ is a finite set of symbols called the alphabet.

• *S* is the set of all finite strings over Σ.

• *G* := (*V*, *E*) is a directed graph consisting of a finite set of vertices and a finite set of labelled edges, *E* ⊆ *V* × *S* × *V.*

The token lattice *G* is constructed for some text *s ε S* as follows:

• Let *L* := {*i* : *i ε* ℕ_0_, 0 ≤ *i* ≤ *length*(*s*)}*.*

• *s′* is a slice of *s*; *s*′ := *s*[*i* : *length*(*s*)] given an *i ε L*.

• *v_i_ ε V* for *i ε L.* These vertices represent a position between characters in *s.*

• For every slice of *s* and corresponding token transducer output *τ*(*c*, *s'*) = (*l*, (*t*_0_, …, *t_m_*)), a path of edges, (*e*_0_, *e*_1_, *…*, *e_m_*), in the token lattice, *G*, is constructed where the first and last vertices of the path correspond to a position between characters, *e*_0_[0] = *v_i_* and *e_m_*[2] = *v_i_*_+_*_l_*, and an edge is associated with a token by *label*(*e_j_*) = *t_j_.*

#### Pseudocode

Of the three described software components, only the lattice constructor’s pseudocode is presented. This is due to token transducer code being specific to a token transducer’s objective and due to existing documentation of a best-path selection algorithm (e.g. [12]).

### A Systematic Approach to Creating a Biomedical Tokenizer

Given our token lattice design pattern, a biomedical tokenizer can be created by:

• Choosing a set of documented POS tags such as the Penn Treebank’s.

• Choosing a best path selection algorithm. Implement the algorithm, if necessary.

• Identifying the token transducers. Implement the transducers, if necessary.

#### Identifying Token Transducers

The proposed tokenizer design pattern does not provide a method for identifying token transducers. Token transducers will vary depending on the tokenizer’s input. For example, the token transducers required for English will likely differ from the token transducers required for Spanish. In this section, we propose a systematic approach to token transducer identification. The guidelines are as follows:

• Select a set of documented POS tags such as the Penn Treebank’s.

• Collect text segments (e.g. sentences) from the input texts that are representative of the input texts’ diversity. This may be via random sampling or another method.

• For each text segment, identify its tokens.

– Adhere to POS tag definitions

– Insure that each token corresponds to at least one POS tag.

– Do not segment text when segmentation results in an unlikely POS-tag sequence such as segmenting “di-trans,poly-cis-Undecaprenyl-diphosphate” into (space separated) “di trans , poly cis Undecaprenyl diphosphate”. This can be captured as *P*(*t_t_|c*, *s*, *L_m_*) >*t* using the introduced notation (the probability of a sequence of POS-tagged tokens given some context, input string and language model is greater than a threshold).

– Segment text when text ambiguously maps to multiple POS tags and segmenting establishes a single POS tag per token (e.g. “2.4kilograms” becomes “2.4” and “kilograms”)

• Categorize the identified tokens into token classes (e.g. “1”, “6.2”, “10 000” and “III” are numerical).

– Base classes on POS tag definitions, named entities (e.g. person, organization, chemical substance), abbreviations and acronyms.

– Minimize the number of classes and multi-class tokens.

• Create a token transducer for each class of token.

#### Example Token Transducer Identification

What follows is an example application of the token transducer guidelines using the Penn Treebank’s POS tag set, an author’s language model and the following sample descriptions:

1. Entire upper dental arch (body structure)

*Segmentation:* Entire upper dental arch ( body structure )

2. Royal Navy - non-commissioned personnel (occupation)

*Segmentation:* Royal Navy - non-commissioned personnel ( occupation )

3. Primidone 50mg tablet

*Segmentation:* Primidone 50 mg tablet

4. Primary Sjogren’s syndrome with organ/system involvement (disorder)

*Segmentation:* Primary Sjogren ’s syndrome with organ and system involvement ( disorder )

5. Posterior cervical spinal cord injury, without spinal injury, C1-4

*Segmentation:* Posterior cervical spinal cord injury , without spinal injury , C1 to 4

6. Precorrin-3B C17-methyltransferase

*Segmentation:* Precorrin-3B C17-methyltransferase

7. Salmonella III arizonae 47:k:1,5,7

*Segmentation:* Salmonella III arizonae 47:k:1,5,7

Item 1 is an example of a simple segmentation.

Item 2 includes two uses of the symbol -. The first use is assigned the POS tag *:* whereas the second use, a hyphen in the token *non-commissioned*, is more difficult to assess. The hyphen could have been removed resulting in two tokens. Since hyphen removal might decrease POS tag sequence likelihood, *non-commissioned* was segmented as one token. For this limited example, either segmentation could be considered acceptable.

The text *50mg* of Item 3 is segmented because segmenting establishes a single POS tag per token. The text would otherwise be a partial match to at least two POS category descriptions. For similar reasons, *C1-4* of Item 5 is segmented into multiple tokens.

The Penn Treebank specifies possessives as a separate POS category. Given this definition, the possessive *’s* is split from *Sjogren’s*.

Items 4, 5, 6 and 7 are segmented to maintain likely POS tag sequences. That is, *47:k:1*,*5*,*7*, *Precorrin-3B* and *C17-methyltransferase* remain as one token, whereas *organ/system* and *C1-4* are modified.

Given these segmentations the resulting token transducers are:

• Alphabetic (dental)

• Possessive (’s)

• Independents (- ,)

• Numeric (50)

• Abbreviations (- for *to* and / for *and*)

• Functional names (C1)

• Substances (Precorrin-3B, C17-methyltransferase, 47:k:1,5,7)

### Testing

We applied the design pattern and the token transducer identification guidelines in the creation of a tokenizer for biomedical concept descriptions and compared our tokenizer to three other tokenizer methods.

#### Test Data

Biomedical concept descriptions were extracted from SNOMED CT [22]. SNOMED CT (Systematized Nomenclature of Medicine – Clinical Terms) is a clinical terminology that contains approximately 387000 concepts, 1.4 million relationships and 1.1 million additional concept descriptions. SNOMED CT is described as a comprehensive clinical terminology, with an objective of “precisely representing clinical information across the scope of health care” [22]. The concept descriptions were extracted from the January 2010 release’s current concepts (as opposed to historical concepts).

We randomly selected 2781 current SNOMED CT concept descriptions to create the ground truth (gold standard) tokenizations. An example concept description is “Posterior cervical spinal cord injury, without spinal injury, C1-4”. An author manually segmented each description by following our definitions and guidelines. He is a native English speaker. A second individual also segmented the concept descriptions after reading instructions and practicing on several examples. The instructions and examples can be found in Appendix . The second individual has a health sciences background but is not a native English speaker.

The second segmentor was provided with open-ended segmenting instructions and five examples. The segmentor read the instructions and segmented the examples, after which the preferred segmentations were presented. This was sufficient for the segmentor to conclude that segmentation “*separated units of meaning*”. The segmentor was encouraged to develop their own segmentation strategy given that this strategy included the two rules provided in the instructions.

The greatest effect of our segmentation definitions and guidelines was to expand closed-class words into their regular form. For example, plus and slash separated lists were converted to regular lists (e.g. “paracetamol + caffeine” became “paracetamol and caffeine”). Similarly, dashes representing the word “to” were replaced (e.g. “C1-4” becomes “C1 to 4”) and slashes representing the word “per” were replaced (e.g. “ml/g” becomes “ml per g”). Knowing that these abbreviated forms were generally absent in the training data, their expansion was to satisfy the requirement of likely POS tag sequences.

Segmentation agreement is presented in Table 1. Agreement was measured with Cohen’s Kappa (CK) [23] - a statistic that accounts for chance agreement. The probability of chance agreement was calculated as 0.5. CK is typically calculated in context of categorical agreement (e.g. POS taggers agree that a word is an adjective). In our case, agreement was defined as both segmentors producing identical segmentations for a given concept description. We modeled chance agreement as a coin toss, where one side of the coin is labeled agree and the other disagree. Thus, for each concept description we could flip our coin to determine whether the segmentations would agree by chance. The expected probability of chance agreement is 0.5.

**Table 1:** Inter-segmentor agreement.

Description	Percent Agreement	Cohen’s Kappa
Preliminary	56.9	0.139
Parentheses corrected	94.4	0.888
Final corrected	95.8	0.916

Inter-segmentor agreement on SNOMED CT concept description segmentations.

There was weak preliminary agreement (CK 0.139) because descriptions ending with a parenthesized word such as “(finding)” were considered one segment by the second segmentor. She judged these parenthesized endings to have a single meaning and thus a single segmentation. (It is interesting to consider that parentheses and punctuation in general have no explicit semantics.) When the second segmentor encountered descriptions ending with several words within parentheses, she opted for segmentation consistency (not separating parentheses) rather than changing completed segmentations (changing single parenthesized words).

An author segmented the parentheses and agreement was recalculated. This single change of separating parentheses from their adjoining words, for words located at the end of concept descriptions, resulted in a CK of 0.888. Further minor corrections to both segmentor’s results such as segmenting missed possessives resulted in a CK of 0.916. The author’s corrected segmentations were adopted for testing. These segmentations appear to be reasonable segmentations given a CK of 0.916 with another segmentor.

#### Tokenizer methods

We constructed a baseline whitespace-only tokenizer and selected tokenizers specifically designed for biomedical text from the list provided by He and Kayaalp [7]. Specialist [24] and Medpost [25] were selected.

Specialist is written in Java. Specialist considers a contiguous run of alpha-numeric characters bounded by white space as a token, as well as individual punctuation. Specialist over-segments and repairs the segmentation into meaningful tokens at a latter stage. For example, “2.4” is tokenized as (space delimited) “2 . 4” and corrected post-tokenization. Specialist was run using the following command: *java -classpath nlpProject.jar gov/nih/nlm/nls/utils/Tokenize –inputType*=*freeText –tokens*.

Medpost is written in C++ and uses 33 interdependent heuristics to tokenize biomedical text. It segments text for further processing which includes POS tagging. Medpost’s POS tag set is based on the Penn Treebank’s POS tag set. Medpost was run using the following command: *medpost -text*.

We implemented the adapted Viterbi algorithm [12] to choose a best-path (tokenization) from the token lattice. We created two variants of the algorithm’s hidden Markov Model (HMM) [1]. These variants were a zero order and first order HMM. The zero order HMM does not employ transitional probabilities whereas the first order does. The first order’s transitional probability relies on one previous state, *P*(*state|state'*)*.*

Our tokenization methods are written in Python (http://www.python.org) and use NLTK (http://www.nltk.org, version 2.0b8) [26], a natural language toolkit library. We trained our HMM’s on a sample (%10) of the Penn Treebank corpus. The sample contains newspaper text.

In one case, we augmented the sample Penn Treebank corpus with %10 of the publicly available MedPost POS tagged corpus [25]. The MedPost corpus contains 6695 sentences from MEDLINE abstracts. Its POS tag set is based on the Penn Treebank’s. We ran a script provided in the MedPost download to convert the MedPost POS tag set to the Penn Treebank’s.

To identify token transducers, we segmented concept descriptions by whitespace and constructed a set from these segmentations. Prior examination of the concept descriptions had shown that whitespace was rarely found within a token. We randomly selected 1900 items from the set of segmentations. These segmentations were separated into tokens by following our guidelines and using the Penn Treebank’s POS tags. Several segmentations were tokenized in context of their associated descriptions because the text segment contained insufficient information to perform tokenization (e.g. the “+” in “Paracetamol + caffeine”). Table 2 summarizes the resulting token classes.

**Table 2:** Token classes derived from SNOMED CT concept descriptions.

Class	Examples
Whitespace	
Independents	[ ? )
Dash or Hyphen	ACHE - Acetylcholine
Alphabetic	Does or dental
Numeric	1500 1.2 10,000 III 1/2
Possessive	’s
Substances	2-chloroaniline
Serotypes	O128:NM
Abbreviations	L.H. O/E
Acronyms	DIY
Lists	Paracetamol + caffeine
Range	C1-4
Functional names	H-987

Token classes derived from SNOMED CT concept descriptions.

#### Accuracy

The tokenizers were applied to our ground truth data (45.5 percent of the data contained ambiguous token boundaries). A segmentation identical to the ground truth’s was considered successful and any other tokenization was considered in error. Table 3 summarizes the results. Medpost and our adapted Viterbi tokenizer performed best with a 92.9% and 92.4% accuracy respectively. Confidence intervals (95% confidence) were calculated using the normal approximation method of the binomial confidence interval [27].

**Table 3:** Tokenizer results.

Tokenizer	Accuracy (%)	Confidence Interval, 95%
Whitespace	53.9	52.0, 55.8
Specialist	47.7	45.8, 49.6
Medpost	92.9	91.9, 93.9
Adapted Viterbi, 0-order HMM	70.8	69.1, 72.5
Adapted Viterbi, 1st-order HMM (AV-1)	84.6	83.3, 85.9
AV-1 + random 10% of MedPost corpus	92.4 (5 run avg)	91.4, 93.4

Tokenizer results.

## Discussion

Specialist performed poorly because it takes a different approach to tokenization, that of over-segment and repair. Specialist also removes symbols from the output tokens, such as brackets, resulting in poorer performance than the baseline whitespace-only tokenizer.

MedPost’s most consistent error was leaving a quantity and its unit joined rather than segmenting them. For example, MedPost would leave “10mg” as a token whereas our approach was to segment “10mg” into “10” and “mg”.

Our most accurate tokenizer’s most consistent error was separating decimal numbers. For example, our algorithm would separate “0.123” into “0 . 123” (space separated). One explanation could be that our training data contained an insufficient quantity of decimal numbers. Unless the HMM had been trained with the decimal number then the token was unknown to our HMM. Training an HMM using token features as well as the token itself would likely improve our most accurate tokenizer.

The adapted Viterbi tokenizer, implemented using our proposed design pattern and our token transducer identification guidelines, performed as well or better than current biomedical text tokenizers. The results suggest that the design pattern and guidelines are a viable alternative to current biomedical tokenization methods.

POS tag sequences and training data have a significant impact on proper text tokenization. The 0-order HMM disregards transition probabilities and consequently POS tag sequences, whereas the 1st-order HMM considers one previous state. Considering one previous state improves tokenization by approximately 15%. A further improvement of approximately 10% is achieved by training the HMM on data that has greater resemblance to the testing data. In other words, ambiguous tokenizations can be disambiguated through POS tagging.

Dividing software into well defined components can increase software extensibility and reuse [28]. Our design pattern should increase tokenizer extensibility and reusability. For example, token transducers can be reused in other token-lattice tokenizers. As an example of extensibility, consider applying a token-lattice tokenizer to new text. This should consist of identifying the new text’s token transducers, including these transducers in the existing tokenizer and possibly training the tokenizer with additional data. This is expected to be less programming work than modifying a large number of segmentation heuristics.

## Conclusions

We presented our tokenizer design pattern named the token lattice design pattern and associated token identification guidelines. We described the tokenizer’s input, output and components. The components are a token lattice and lattice constructor, a best lattice-path chooser and token transducers. Our evaluation of our design pattern and guidelines supports our claim that the design pattern and guidelines are a viable approach to tokenization. The token lattice design pattern is expected to apply to domains other than the biomedical domain.

Our evaluation demonstrates that ambiguous tokenizations can be disambiguated through POS tagging. In doing so, POS tag sequences and training data have a significant impact on proper text tokenization. Our approach of tokenization through POS tagging differs from previous split-join classification approaches.

Our tokenizer formalization suggests how various biomedical text processing components such as machine learning of named entities can interact cooperatively (as token transducers). Our formalization also demonstrates that machine learning algorithms are appropriate for choosing the best-lattice path from a (biomedical text) token lattice.

Our research results support further investigation of machine learning on token lattices for selecting the best-lattice path. Future work includes applying the tokenizer pattern to other biomedical texts (e.g. palliative care consult letters) and testing new best lattice-path chooser algorithms. Improvements to token transducers and the best lattice-path chooser are expected to further improve tokenization.

## Competing Interests

The authors declare that they have no competing interests

## Authors contributions

NB is a PhD student at the University of Victoria. This work has been created as part of his PhD research. JWJ is a faculty member at the university and NB’s supervisor.

## Appendix - Secondary Segmentor Instructions

You are asked to segment a sentence into its tokens (pieces). Here’s an example (sentence followed by tokens, one per line):

A car, faster than lighting, was painted red.

A

car

,

faster

than

lighting

,

was

painted

red

When segmenting a sentence you are permitted to 1) separate and 2) delete pieces of the sentence. In the example above, spaces were deleted and punctuation was separated from its adjoining word.

Tokens may have spaces (whitespace). Some people *may* choose to do the following:

New York is a big city.

New York

is

a

big

city

Below are segmenting rules that you must follow, These rules apply to very few situations. For most cases, you will decide how to segment a sentence.

• Consider the following as separate tokens (upper or lower case): ’ll ’re ’ve n’t ’s ’

• Abbreviations of closed-class words must be expanded. Example: The sentence ”Jon/Roger are running.” would become ”Jon and Roger are running.” Here is a list of closed-class words: a about above across after against all along although among an and another any anybody anyone anything around as at because before behind below beneath beside between beyond both but by despite down during each either enough ever every everybody everyone everything except few for from he her hers herself him himself his how i if in inside into it its itself like many me mine myself near neither no nobody none nor of off on once one onto or ours ourselves out outside over past per several she since so some somebody someone sufficient than that the theirs them themselves these they this those though through throughout till to toward under underneath until up upon us we what whatever when where whether which whichever while who whoever whom whomever with within without yet you yours yourself yourselves

Apply what you’ve just learned to these examples:

Entire upper dental arch (body structure)

Entire

upper

dental

arch

(

body

structure

)

Royal Navy - non-commissioned personnel (occupation)

Royal

Navy

-

non-commissioned

personnel

(

occupation

)

Posterior cervical spinal cord injury, without spinal injury, C1-4

Posterior

cervical

spinal

cord

injury

,

without

spinal

injury

,

c1

to

4

Primidone 50mg tablet

Primidone

50

mg

tablet

Precorrin-3B C17-methyltransferase

Precorrin-3B

C17-methyltransferase
